# Relevance of investigating light transmittance through red protective shields in dentistry

**DOI:** 10.1038/bdjopen.2018.4

**Published:** 2018-05-04

**Authors:** Ellen Bruzell, Bjørn Johnsen, Terje Christensen

**Affiliations:** 1 Nordic Institute of Dental Materials, Oslo, Norway; 2 Norwegian Radiation Protection Authority, Østerås, Norway

Thank you for the high-quality content of the *BDJ* and *BDJ Open*. However, we became aware of a recent publication that did not represent the usual up-to-date information in the field of dentistry, namely ‘A study on light transmittance through red protective shields modified with different window films’ published on 30 June 2017 in *BDJ Open*.^1^ Although we are convinced that the authors have the best intentions in conveying the message that clinicians’ eyes must be protected during treatments using optical radiation, we are afraid that a solution is presented to a marginal or non-existent problem. As the wavelengths emitted by LED-based dental light-curing devices so closely match those that are most detrimental to retina and the output of these lamps are high enough to theoretically exceed the limit values in normal use within a workday, the focus should be on eye protection against blue light.

In evaluating the risk of eye hazards due to optical radiation the following questions must be addressed: What is the source and magnitude of exposure? Which type of hazards can be induced? What is the exposure limit? To our knowledge, most dental light-curing devices being produced today are LED-based and these do not emit any red or infrared radiation. There are likely to be some halogen lamps still in use, some of which may not be in accordance with the standard for dental light-curing devices (‘Powered polymerization activators’, ISO 10650:2015). Radiation outside the absorption spectrum of the most commonly used photoinitiators in the material to be cured is neither needed nor justified, and optical filters are often used to remove light of these unwanted wavelength ranges. In the absence of such filters some of these devices may theoretically emit red or near-infrared radiation. According to the ISO standard there is a maximum level for the emission of wavelengths above 515 nm (green light and longer wavelengths). When lamps comply with this standard it is highly unlikely that wavelengths outside the violet–blue range pose any threat for photochemical retinal hazard in normal use.

Another potential eye hazard due to optical radiation is heat. Thermal damage to the retina and iris may be induced by visible light and near-infrared radiation. However, the output of the red and near-infrared wavelengths is not high enough to exceed the exposure limit values (International Commission on Non-Ionizing Radiation Protection. ICNIRP Guidelines on Limits of Exposure to Incoherent Visible and Infrared Radiation, 2013). During the ~15 years we have measured and evaluated spectral data of about 50 different lamp systems intended for use in the dental clinic, we observed only two lamps that emitted any radiation (1% of maximum value) above 700 nm (‘border’ visible/near-infrared) and those lamps were specifically made for tooth bleaching purposes.

Still, the radiation output was too low to induce thermal damage to the eyes of the patient or operator in normal use. Furthermore, judging protection efficiency from the colour of the protective filters is not an accurate measure of transmittance and does not guarantee adequate protection.

According to our calculations, the transmittance level should be below 0.1% in the blue light wavelength range. After measuring the transmittance of 30 commercially available orange filters, we found that about half of them were inferior as eye protection against reflected blue light.
